# Polydatin inhibits growth of lung cancer cells by inducing apoptosis and causing cell cycle arrest

**DOI:** 10.3892/ol.2013.1696

**Published:** 2013-11-21

**Authors:** YUSONG ZHANG, ZHIXIANG ZHUANG, QINGHUI MENG, YANG JIAO, JIAYING XU, SAIJUN FAN

**Affiliations:** 1 Department of Oncology, The Second Affiliated Hospital of Soochow University, Suzhou, Jiangsu 215004, P.R. China; 2 School of Radiation Medicine and Protection, Medical College of Soochow University, Suzhou, Jiangsu 215123, P.R. China; 3 Department of Oncology, Lombardi Comprehensive Cancer Center, Georgetown University, Washington, DC 20057, USA

**Keywords:** polydatin, lung cancer, apoptosis, cyclin D1

## Abstract

Polydatin (PD), a small natural compound from *Polygonum cuspidatum*, has a number of biological functions. However, the anticancer activity of PD has been poorly investigated. In the present study, thiazolyl blue tetrazolium bromide assay was used to evaluate the inhibitory effect of PD on cell growth. Cell cycle distribution and apoptosis were investigated by flow cytometry. In addition, the expression of several proteins associated with apoptosis and cell cycle were analyzed by western blot analysis. The results demonstrated that PD significantly inhibits the proliferation of A549 and NCI-H1975 lung cancer cell lines and causes dose-dependent apoptosis. Cell cycle analysis revealed that PD induces S phase cell cycle arrest. Western blot analysis showed that the expression of Bcl-2 decreased as that of Bax increased, and the expression of cyclin D1 was also suppressed. The results suggest that PD has potential therapeutic applications in the treatment of lung cancer.

## Introduction

Lung cancer is a growing global health problem and has become the most common type of cancer that results in mortality in males and females in developed countries (1). Therapeutic strategies include surgery, radiotherapy, chemotherapy, and targeted and combined therapies. Despite advances in treatment, non-small cell lung cancer, which accounts for 80–85% of all cases of lung cancer (2), remains an aggressive lung cancer with poor patient survival rates. To date, chemotherapy has been the most frequently used therapeutic strategy for lung cancer in advanced stages. However, the outcome of chemotherapy in patients with advanced lung cancer is poor. The median survival rate of advanced lung cancer patients treated with standard platinum-based chemotherapy is ~10 months (3). Thus, a novel agent for lung cancer therapy is continually being investigated.

With developments in phytochemistry, an increasing number of individuals are acknowledging the importance of herbal plants. Among the 155 small molecular anticancer drugs developed between the 1940s and June 2006, 47% are natural products or their derivatives (4). Examples of plant-based therapeutic anticancer drugs are camptothecin from *Camptotheca acuminate*, etoposide from *Podophyllum peltatum*, vincristine from *Catharanthus roseus* and paclitaxel from yews of the genus *Taxus*(5,6).

**Figure 1:**
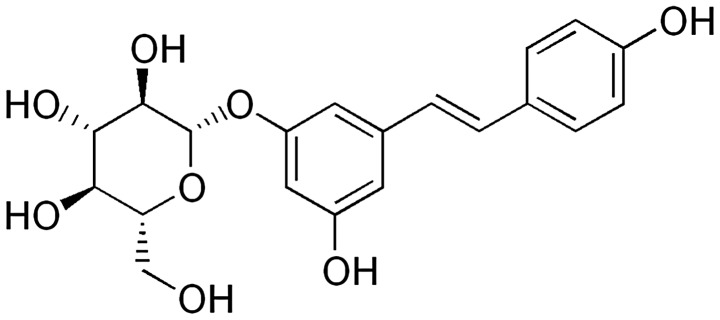
Chemical structure of polydatin.

*Polygonum cuspidatum*, a traditional Chinese medicinal herb commonly used for its root and rhizome, has been officially listed in the Pharmacopoeia for a number of years. 3,4,5′-Trihydroxystilbene-3-β-D-mono-D-glucoside [polydatin (PD)], the chemical structure of which is shown in Fig. 1, is one of the main effective elements of *P. cuspidatum*. Previously, pharmacological studies and clinical practice have demonstrated that PD has a number of biological functions, such as protective effects against shock (7–9), ischemia/reperfusion injury (10,11), congestive heart failure (12) and endometriosis (13). However, few previous studies have analyzed the effects of PD on human cancer cells. In the present study, the effects of PD on the proliferation, cell cycle phase distribution and apoptosis of human A549 and NCI-H1975 lung adenocarcinoma cancer cell lines and potential mechanisms were investigated.

## Materials and methods

### Chemicals

LKT Laboratories, Inc. (St Paul, MN, USA) was the supplier of the PD (cat. no. P5845) used. PD was dissolved in a stock solution of 10 mmol/l dimethysulfoxide (DMSO) and directly diluted in medium to appropriate concentrations prior to the experiments. Thiazolyl blue tetrazolium bromide (MTT; cat. no. M2128) was purchased from Sigma-Aldrich (St. Louis, MO, USA) and Annexin V-conjugated Alexa Fluor 488 apoptosis detection kits (V-13245) were obtained from Molecular Probes, Inc. (Eugene, OR, USA). Primary antibodies against Bcl-2, Bax and cyclin D1 and secondary antibodies were purchased from Santa Cruz Biotechnology, Inc. (Santa Cruz, CA, USA). The Bio-Rad protein assay kit II was supplied by Bio-Rad (Hercules, CA, USA) and the enhanced chemiluminescent western blot detection reagents (cat. no. RPN2106) were obtained from Amersham Pharmacia Biotech (Amersham, UK).

### Cell lines and cell culture

Cancer cell lines were purchased from American Type Culture Collection (Manassas, VA, USA). The cells were maintained as a monolayer in DMEM or RPMI-1640 medium supplemented with 10% fetal calf serum, 2 mmol/l glutamine, 100 μg/ml streptomycin and 100 U/ml penicillin, in a humidified atmosphere containing 5% CO_2_. Cells in the logarithmic phase were used in the experiments.

### MTT viability assay

Determination of cell viability was performed using an MTT assay as described previously (14). Briefly, cells were incubated in flat-bottom, 96-well plates (6×10^3^ cells/well) overnight. Then, cells were treated with DMSO (0.1%) or an increasing dosage of PD. Following 20, 44 and 68 h of treatment, 20 μl MTT (5 mg/ml) was added to each well and further incubated for 4 h. Cells were then solubilized in 150 μl DMSO. The absorbance reading was obtained using a Dynatech 96-well spectrophotometer (Dynatech Laboratories, Chantilly, VA, USA). The amount of MTT dye reduction was calculated based on the difference between the absorbances at 570 and 630 nm. The cell viability in treated cells was expressed as the amount of dye reduction relative to that of the untreated control cells.

### Apoptosis assays and cell cycle distribution analysis

The percentage of cells that actively underwent apoptosis was analyzed using Annexin V-phycoerythrin-based immunofluorescence, as described previously (15). Briefly, the cells were incubated in six-well plates (2.5×10^5^ cells/well) overnight. The cells were then treated with DMSO or PD for 48 h. Adherent and floating cells were collected, washed in cold phosphate-buffered saline (PBS) twice and stained with Annexin V-PE, according to the manufacturer’s instructions. Cells were identified using a FACSCalibur flow cytometer (BD Biosciences, San Jose, CA, USA). Cells for cell cycle analysis were washed once with PBS and fixed in 70% cold ethanol for ≥4 h. The fixed cells were then washed twice with PBS and resuspended in 500 μl propidium iodide (10 mg/ml) containing 300 μg/ml RNase. Cell cycle distribution was calculated from 10,000 cells with ModFit LT software (Verity Software House, Topsham, ME, USA), using FACSCalibur.

### Western blot analysis

Western blot analyses were performed as described previously (16). Cells were treated with DMSO (0.1%) or PD and, following 48 h of treatment, were harvested and lysed. The protein concentration in the lysates was quantified using Bio-Rad Protein Assay reagent (Bio-Rad) following the manufacturer’s instructions. An equal amount of protein was separated by electrophoresis on SDS-polyacrylamide gels (Bio-Rad) and transferred to PVDF membranes (Santa Cruz Biotechnology, Inc.). Following blocking with 5% non-fat milk, the membranes were incubated with the desired primary antibodies overnight at the following dilutions: Anti-Bcl-2, 1:500; anti-Bax, 1:1,000; anti-cyclin D1, 1:1,000; and anti-β-actin, 1:20,000. Subsequently, the membranes were incubated with appropriate secondary antibodies. The immunoreactive bands were visualized using enhanced chemiluminescence, according to the manufacturer’s instructions.

### Statistical analysis

Data are presented as the mean ± standard deviation. Statistical analysis was performed by multifactorial analysis of variance using SPSS software (SPSS, Inc., Chicago, IL, USA). P<0.05 was considered to indicate a statistically significant difference.

## Results

### PD has a wide anticancer spectrum and is more potent in eliminating cancer cells than non-cancer cells

**Figure 2:**
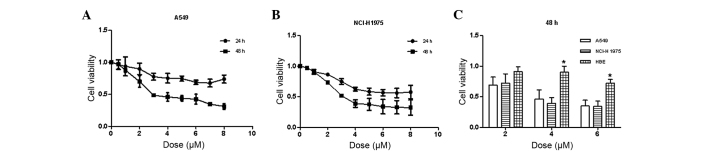
Inhibitory effects of PD on the growth of human lung cancer and non-cancer HBE cells. Exponentially growing cells in 96-well plates were continuously treated with the indicated concentrations of PD for 24 and 48 h and then subjected to MTT viability assay. Dose-response curves of PD in (A) A549 and (B) NCI-H1975 cells 24 or 48 h following treatment. (C) Viability of A549, NCI-H1975, and HBE cells 48 h following 2, 4, and 6 μM PD treatment. Data are presented as the percentage of DMSO-treated controls (mean ± SD) from three independent experiments. PD, polydatin; HBE, human bronchial epithelial; MTT, thiazolyl blue tetrazolium bromide; DMSO, dimethysulfoxide.

The cytotoxicity of PD in 10 cancer cell lines was first determined by MTT assay. The decrease in absorbance in this assay was due to cell death or reduction in cell proliferation. As shown in Table I, PD exhibits broad-spectrum growth inhibition against 10 cancer cell lines. A dose-dependent and time-dependent inhibition of human lung cancer cells was shown (Figs. 2A and B). By comparing the effects of PD in inhibiting cell growth between cancer and non-cancer cells (Fig. 2C), it was found that 6 μmol/l PD caused 65% (48 h) loss of cell viability in A549 lung cancer cells and 66% (48 h) loss in NCI-H1975 lung cancer cells. However, at the same concentration, loss of cell viability in human bronchial epithelial (HBE) cells derived from normal HBE cells was 28% (48 h). This result indicated that PD is more potent in eliminating cancer cells than non-cancer cells.

### PD induces apoptosis in lung cancer cells

**Figure 3:**
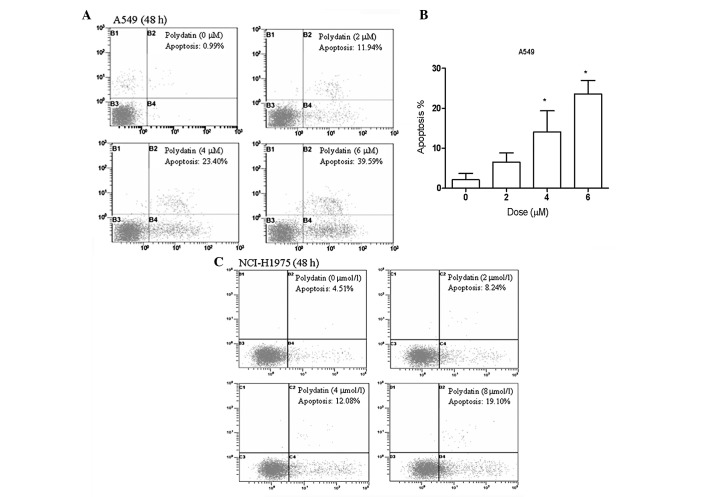
Apoptotic effects of PD on A549 and NCI-H1975 human lung cancer cells. Cells were treated with the indicated concentrations of PD for 48 h, stained with Annexin-V-PE and then analyzed for apoptosis by DNA flow cytometry. The results indicate the percentage of Annexin V-positive cells (apoptosis). All experiments were performed in duplicate and yielded similar results. (A) Original images of apoptosis in A549 cells. (B) The percentage of cells undergoing apoptotic cell death is presented as the mean ± SD from three separate experiments in A549 cells. ^*^P<0.05, vs. the respective controls. (C) Original images of apoptosis in NCI-H1975 cells. PD, polydatin.

To investigate the features of PD-induced lung cancer cell growth inhibition, A549 and NCI-H1975 lung cancer cells were treated with various concentrations of PD for 48 h. Subsequently, apoptosis was detected by flow cytometry. As shown in Figs. 3A and B, PD activates apoptosis in A549 lung cancer cells in a dose-dependent manner. The percentage of cells undergoing apoptotic cell death increased from 0.99% in the control culture to 39.5% following exposure to 6 μmol/l PD for 48 h in A549 lung cancer cells. Similar results were observed in NCI-H1975 cell lines (Fig. 3C).

### PD induces S-phase cell cycle arrest in lung cancer cells

**Figure 4:**
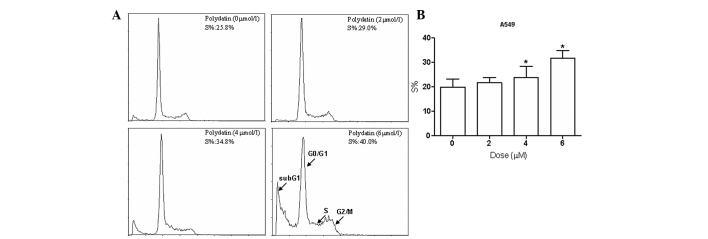
Cell cycle arrest induced by PD in A549 human lung cancer cells. Cells were treated with increasing concentrations of PD. Following 48 h of treatment, cells were labeled with propidium iodide and then analyzed by DNA flow cytometry. (A) Results indicate the percentage of cells in each phase of the cell cycle and sub-G1. All experiments were performed in duplicate and yielded similar results. Original images of cell cycle distribution in A549 cells are presented. (B) The percentage of cells in S phase is presented as the mean ± SD from three various experiments in A549 cells. ^*^P<0.05, compared with the respective controls. PD, polydatin.

To determine whether interference with cell cycle progression is mediated by the PD-based growth inhibition of lung cancer cells, the effects of PD on cell cycle progression were examined in an exponentially dividing culture of A549 and NCI-H1975 cells. The treatment of cells with varying concentrations of PD for 48 h resulted in the increased accumulation of cells in the S phase and a corresponding decrease in the G0/G1 and G2/M phases. PD at a concentration of 6 μmol/l increased the S phase population from 19.91±2.34 to 31.71±1.83% in A549 cells and from 21.41±8.72 to 36.37±3.56% in NCI-H1975 cells (Fig. 4). The typical flow histogram of sub-G1 apoptotic peaks was also detected.

### PD downregulates Bcl-2 and cyclin D1 and upregulates Bax expression in lung cancer cell lines

**Figure 5:**
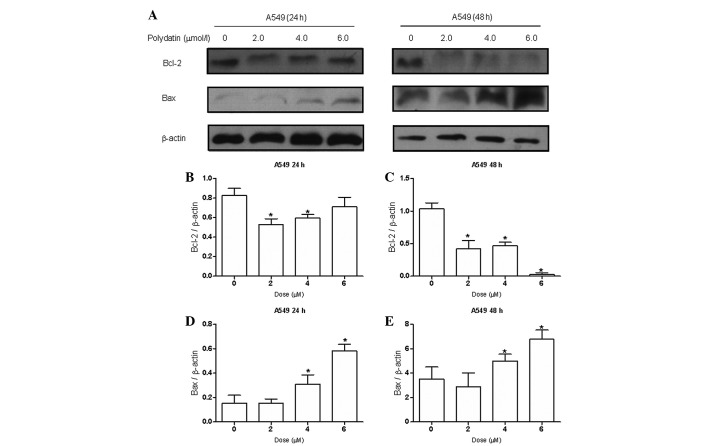
Effect of PD on the expression of apoptosis-related proteins. (A) A549 human lung cancer cells were treated with increasing concentrations of PD for 24 and 48 h. Expression levels of Bcl-2 and Bax were detected by western blot analysis and β-actin was used as a control. (B and C) Bcl-2 and (D and E) Bax protein bands were quantitated by densitometry, and the data are presented as the mean ± SD from three experiments. ^*^P<0.05, compared with the control. PD, polydatin.

**Figure 6:**
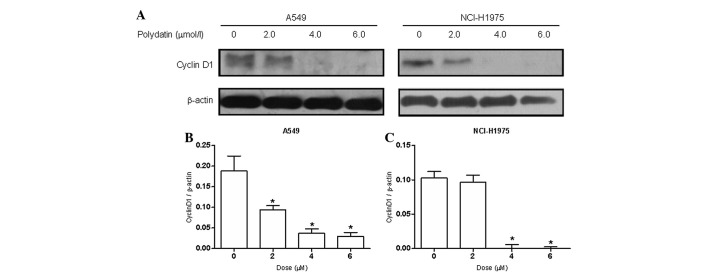
Effect of PD on cyclin D1 expression. (A) A549 and NCI-H1975 human lung cancer cells were treated with increasing concentrations of PD for 48 h. Expression levels of cyclin D1 were detected by western blot analysis and β-actin was used as a control. (B and C) Cyclin D1 protein bands were quantitated by densitometry and the data are presented as the mean ± SD from three experiments. ^*^P<0.05, compared with the control. PD, polydactin.

Due to the effects of PD on apoptosis, the impact of PD on the expression of Bcl-2 and Bax, two key apoptosis regulatory proteins, were examined by western blot analysis. The results indicated (Fig. 5) that PD dose-dependently downregulated the expression of antiapoptotic protein Bcl-2 and upregulated the expression of proapoptotic protein Bax. Following treatment with 6 μmol/l PD, the Bax/Bcl-2 ratio, which favors apoptosis (17), increased significantly in the A549 cells. To explore the mechanism of the effects of PD on S phase cell cycle arrest, the expression levels of cell cycle-related protein cyclin D1 were examined. The results showed (Fig. 6) that the expression of protein cyclin D1 decreased significantly following the treatment of A549 and NCI-H1975 cells with PD for 48 h.

## Discussion

PD is a glycoside of resveratrol, in which the glycoside group is bonded in the C-3 position, substituting a hydroxyl group. This substitution leads to conformational changes in the molecule, resulting in changes in its biological properties. PD is more efficiently absorbed (18,19) and more resistant to enzymatic oxidation than resveratrol (20) and is soluble in hot water. In contrast to resveratrol, which penetrates cells passively, PD enters cells via an active mechanism using glucose carriers (21). These properties provide PD with greater bioavailability than resveratrol.

Previous studies have demonstrated the chemopreventive and anticancer activities of resveratrol (22–31). However, little is known concerning the antitumor activity of PD. For the first time, the current study examined the cytotoxic effect of PD in various cancer cell lines and PD was found to have potent growth inhibitory effects on leukemia, breast, lung, cervical, ovarian, liver and nasopharyngeal cancer cells. In particular, PD had less toxicity to non-neoplastic HBE cells. This suggests that PD may be a potent chemotherapeutic agent.

Apoptosis, also known as programmed cell death, is morphologically characterized by cell shrinkage, membrane remodeling, cell blebbing, chromatin condensation and DNA fragmentation with apoptotic bodies (32). Apoptosis activation is considered to be a good target in cancer therapies (33,34). A number of anticancer drugs act through the induction of apoptosis to prevent tumor promotion and progression. In general, apoptosis is regulated by proapoptotic and antiapoptotic proteins of the Bcl-2 family, and is executed through caspases (or cysteine-aspartic proteases). The induction of apoptosis in tumor cells has been proposed to result from the inability of Bcl-2 to form heterodimers with Bax. Bax overexpression increases the sensitivity of cells to anticancer drugs due to the lack of Bcl-2 in the cell. An increase in the ratio of Bax/Bcl-2 stimulates the release of cytochrome *c* from the mitochondria into the cytosol, which leads to the activation of caspase-3 (35,36). The results of the present study showed that PD induces apoptosis in lung cancer cells effectively. The induction of apoptosis was accompanied by an increase in Bax expression and a decrease in Bcl-2 expression. The results support the development of PD for lung cancer prevention and treatment.

The control of cell cycle progression in cancer cells is a potentially effective strategy to arrest tumor growth (37,38). Cyclin D1, an important regulator of cell cycle progression, functions as a transcriptional coregulator (39). Overexpression of cyclin D1 has been described in a wide spectrum of human cancer types, such as breast, lung, liver and brain cancer (40–42). Cyclin D1 levels must be high during the G1 phase to initiate DNA synthesis, but must be suppressed to low levels during the S phase for efficient DNA synthesis. To continue cell proliferation, cyclin D1 must be induced once again during the G2 phase (43). The *in vitro* results of the current study indicated that the treatment of A549 and NCI-H1975 cells with PD results in the S-phase arrest of cell cycle progression. Western blot analysis showed that the expression level of cyclin D1 was inhibited, whereas, cyclin A, B1 and E expression levels were not affected (data not shown). These results suggest that PD inhibits the proliferation of cancer cells by inhibition of cyclin D1 expression, thereby, reducing cell cycle progression by arresting the cells at S phase.

The present study performed a preliminary investigation of the inhibitory effect of PD on lung cancer cells. The antiproliferation effect of PD involves the suppression of cell cycle progression and induction of apoptosis in human lung cancer cells. Apoptosis was initiated by upregulating Bax levels together with downregulating Bcl-2 levels. However, the anti-tumor effect and toxicity of PD *in vivo* is unknown. Future studies on the *in vivo* effect of PD are necessary. Current investigations on the mechanism and the *in vivo* anticancer efficacy of PD are in progress.

## Figures and Tables

**Table I:** IC_50_ values of PD in various cancer cell lines.

Cell lines	IC_50_ (48 h)
Lung cancer	
A549	2.95±0.37
NCI-H1975	3.23±0.46
Breast cancer	
MDA-MB-231	2.66±0.73
MCF-7	1.49±0.26
Cervical cancer	
Hela	2.13±0.52
Ovarian cancer	
SKOV-3	4.44±0.89
Liver cancer	
SMMC-7721	2.43±0.27
Nasopharyngeal cancer	
CNE-1	5.62±1.28
Leukemia	
HL-60	1.63±0.91
K562	1.91±0.37

PD, polydatin.

## References

[1] Spiro SG, Tanner NT, Silvestri GA (2010). Lung cancer: progress in diagnosis, staging and therapy. Respirology.

[2] Stinchcombe TE, Fried D, Morris DE, Socinski MA (2006). Combined modality therapy for stage III non-small cell lung cancer. Oncologist.

[3] Luan J, Duan H, Liu Q, Yagasaki K, Zhang G (2010). Inhibitory effects of norcantharidin against human lung cancer cell growth and migration. Cytotechnology.

[4] Newman DJ, Cragg GM (2007). Natural products as sources of new drugs over the last 25 years. J Nat Prod.

[5] Cragg GM, Newman DJ (2005). Plants as a source of anti-cancer agents. J Ethnopharmacol.

[6] Gordaliza M (2007). Natural products as leads to anticancer drugs. Clin Transl Oncol.

[7] Zhao KS, Jin C, Huang X (2003). The mechanism of Polydatin in shock treatment. Clin Hemorheol Microcirc.

[8] Wang X, Song R, Chen Y, Zhao M, Zhao KS (2013). Polydatin - a new mitochondria protector for acute severe hemorrhagic shock treatment. Expert Opin Investig Drugs.

[9] Wang X, Song R, Bian HN, Brunk UT, Zhao M, Zhao KS (2012). Polydatin, a natural polyphenol, protects arterial smooth muscle cells against mitochondrial dysfunction and lysosomal destabilization following hemorrhagic shock. Am J Physiol Regul Integr Comp Physiol.

[10] Cheng Y, Zhang HT, Sun L (2006). Involvement of cell adhesion molecules in polydatin protection of brain tissues from ischemia-reperfusion injury. Brain Res.

[11] Miao Q, Wang S, Miao S, Wang J, Xie Y, Yang Q (2011). Cardioprotective effect of polydatin against ischemia/reperfusion injury: roles of protein kinase C and mito K(ATP) activation. Phytomedicine.

[12] Gao JP, Chen CX, Gu WL, Wu Q, Wang Y, Lü J (2010). Effects of polydatin on attenuating ventricular remodeling in isoproterenol-induced mouse and pressure-overload rat models. Fitoterapia.

[13] Indraccolo U, Barbieri F (2010). Effect of palmitoylethanolamide-polydatin combination on chronic pelvic pain associated with endometriosis: preliminary observations. Eur J Obstet Gynecol Reprod Biol.

[14] Fan S, Wang JA, Yuan RQ (1998). BRCA1 as a potential human prostate tumor suppressor: modulation of proliferation, damage responses and expression of cell regulatory proteins. Oncogene.

[15] Vermes I, Haanen C, Steffens-Nakken H, Reutelingsperger C (1995). A novel assay for apoptosis. Flow cytometric detection of phosphatidylserine expression on early apoptotic cells using fluorescein labelled Annexin V. J Immunol Methods.

[16] Fan S, Gao M, Meng Q (2005). Role of NF-kappaB signaling in hepatocyte growth factor/scatter factor-mediated cell protection. Oncogene.

[17] Xu JY, Meng QH, Chong Y (2012). Sanguinarine inhibits growth of human cervical cancer cells through the induction of apoptosis. Oncol Rep.

[18] Hollman PC, de Vries JH, van LSD, Mengelers MJ, Katan MB (1995). Absorption of dietary quercetin glycosides and quercetin in healthy ileostomy volunteers. Am J Clin Nutr.

[19] Paganga G, Rice-Evans CA (1997). The identification of flavonoids as glycosides in human plasma. FEBS Lett.

[20] Regev-Shoshani G, Shoseyov O, Bilkis I, Kerem Z (2003). Glycosylation of resveratrol protects it from enzymic oxidation. Biochem J.

[21] Krasnow MN, Murphy TM (2004). Polyphenol glucosylating activity in cell suspensions of grape (*Vitis vinifera*). J Agric Food Chem.

[22] Jang M, Cai L, Udeani GO (1997). Cancer chemopreventive activity of resveratrol, a natural product derived from grapes. Science.

[23] Joe AK, Liu H, Suzui M, Vural ME, Xiao D, Weinstein IB (2002). Resveratrol induces growth inhibition, S-phase arrest, apoptosis, and changes in biomarker expression in several human cancer cell lines. Clin Cancer Res.

[24] Rezk YA, Balulad SS, Keller RS, Bennett JA (2006). Use of resveratrol to improve the effectiveness of cisplatin and doxorubicin: study in human gynecologic cancer cell lines and in rodent heart. Am J Obstet Gynecol.

[25] Liu PL, Tsai JR, Charles AL (2010). Resveratrol inhibits human lung adenocarcinoma cell metastasis by suppressing heme oxygenase 1-mediated nuclear factor-kappaB pathway and subsequently downregulating expression of matrix metalloproteinases. Mol Nutr Food Res.

[26] Shibata MA, Akao Y, Shibata E (2007). Vaticanol C, a novel resveratrol tetramer, reduces lymph node and lung metastases of mouse mammary carcinoma carrying p53 mutation. Cancer Chemother Pharmacol.

[27] Liu HS, Pan CE, Yang W, Liu XM (2003). Antitumor and immunomodulatory activity of resveratrol on experimentally implanted tumor of H22 in Balb/c mice. World J Gastroenterol.

[28] Zhou HB, Chen JJ, Wang WX, Cai JT, Du Q (2005). Anticancer activity of resveratrol on implanted human primary gastric carcinoma cells in nude mice. World J Gastroenterol.

[29] Pan MH, Gao JH, Lai CS (2008). Antitumor activity of 3,5,4′-trimethoxystilbene in COLO 205 cells and xenografts in SCID mice. Mol Carcinog.

[30] Li T, Fan GX, Wang W, Li T, Yuan YK (2007). Resveratrol induces apoptosis, influences IL-6 and exerts immunomodulatory effect on mouse lymphocytic leukemia both in vitro and in vivo. Int Immunopharmacol.

[31] Chen JC, Chen Y, Lin JH, Wu JM, Tseng SH (2006). Resveratrol suppresses angiogenesis in gliomas: evaluation by color Doppler ultrasound. Anticancer Res.

[32] Wyllie AH (1997). Apoptosis: an overview. Br Med Bull.

[33] Neto CC, Amoroso JW, Liberty AM (2008). Anticancer activities of cranberry phytochemicals: an update. Mol Nutr Food Res.

[34] Kaur M, Agarwal R (2007). Transcription factors: molecular targets for prostate cancer intervention by phytochemicals. Curr Cancer Drug Targets.

[35] Yang J, Liu X, Bhalla K (1997). Prevention of apoptosis by Bcl-2: release of cytochrome c from mitochondria blocked. Science.

[36] Kluck RM, Bossy-Wetzel E, Green DR, Newmeyer DD (1997). The release of cytochrome c from mitochondria: a primary site for Bcl-2 regulation of apoptosis. Science.

[37] Graña X, Reddy EP (1995). Cell cycle control in mammalian cells: role of cyclins, cyclin dependent kinases (CDKs), growth suppressor genes and cyclin-dependent kinase inhibitors (CKIs). Oncogene.

[38] Pavletich NP (1999). Mechanisms of cyclin-dependent kinase regulation: structures of Cdks, their cyclin activators, and Cip and INK4 inhibitors. J Mol Biol.

[39] Alao JP (2007). The regulation of cyclin D1 degradation: roles in cancer development and the potential for therapeutic invention. Mol Cancer.

[40] Gillett C, Smith P, Gregory W (1996). Cyclin D1 and prognosis in human breast cancer. Int J Cancer.

[41] Hall M, Peters G (1996). Genetic alterations of cyclins, cyclin-dependent kinases, and Cdk inhibitors in human cancer. Adv Cancer Res.

[42] Molenaar JJ, Ebus ME, Koster J (2008). Cyclin D1 and CDK4 activity contribute to the undifferentiated phenotype in neuroblastoma. Cancer Res.

[43] Yang K, Hitomi M, Stacey DW (2006). Variations in cyclin D1 levels through the cell cycle determine the proliferative fate of a cell. Cell Div.

